# Pivoting school health and nutrition programmes during COVID-19 in low- and middle-income countries: A scoping review

**DOI:** 10.7189/jogh.14.05006

**Published:** 2024-01-19

**Authors:** Bianca Carducci, Georgia Dominguez, Emily Kidd, Karlie Janes, Aatekah Owais, Zulfiqar A Bhutta

**Affiliations:** 1 Centre for Global Child Health, Hospital for Sick Children, Peter Gilgan Centre for Research, and Learning (PGCRL), Toronto, Canada; 2 Department of Nutritional Sciences, Faculty of Medicine, University of Toronto, Toronto, Canada; 3 Centre of Excellence in Women, and Child Health, Aga Khan University, Karachi, Pakistan; 4 Dalla Lana School of Public Health, University of Toronto, Toronto, Canada

## Abstract

**Background:**

Preventive and promotive interventions delivered by schools can support a healthy lifestyle, positive development, and well-being in children and adolescents. The coronavirus disease 2019 (COVID-19) pandemic presented unique challenges to school health and nutrition programmes due to closures and mobility restrictions.

**Methods:**

We conducted a scoping review to examine how school health and nutrition programmes pivoted during the COVID-19 pandemic, and to provide summative guidance to stakeholders in strategic immediate and long-term response efforts. We searched MEDLINE, Embase, PsycINFO, and grey literature sources for primary (observational, intervention, and programme evaluations) and secondary (reviews, best practices, and recommendations) studies conducted in low- and middle-income countries from January 2020 to June 2023. Programmes that originated in schools, which included children and adolescents (5–19.9 years) were eligible.

**Results:**

We included 23 studies in this review. They varied in their adaptation strategy and key programmatic focus, including access to school meals (n = 8), health services, such as immunisations, eye health, and water, sanitation, and hygiene-related activities (n = 4), physical activity curriculum and exercise training (n = 3), mental health counselling and curriculum (n = 3), or were multi-component in nature (n = 5). While school meals, physical activity, and mental health programmes were adapted by out-of-school administration (either in the community, households, or virtually), all health services were suspended indefinitely. Importantly, there was an overwhelming lack of quantitative data regarding modified programme coverage, utilisation, and the impact on children and adolescent health and nutrition.

**Conclusions:**

We found limited evidence of successful adaptation of school health and nutrition programme implementation during the pandemic, especially from Asia and Africa. While the adoption of the World Health Organization health-promoting school global standards and indicators is necessary at the national and school level, future research must prioritise the development of a school-based comprehensive monitoring and evaluation framework to track key indicators related to both health and nutrition of school-aged children and adolescents.

Nutrition during school years is crucial for the physical, mental, and psychosocial development of children and adolescents aged five to 19 years [1]. In 2016, the International Commission on Financing Global Education Opportunity listed health and nutrition interventions as an essential strategy for optimising learning outcomes [2]. However, the temporary but prolonged closure of educational institutions in over 194 countries due to the global coronavirus disease 2019 (COVID-19) pandemic in 2020 has indisputably impacted children and adolescents globally. The United Nations Educational, Scientific, and Cultural Organization estimates that over 90% of the world’s students were out of school during this time, representing 1.54 billion children and youth enrolled in school or university, including nearly 743 million girls [3]. Importantly, given the inequities in access to remote learning opportunities and the important role schools play in the health, nutrition, and well-being of students from vulnerable backgrounds, these temporary school closures are likely to have devastating long-term impacts on children’s health, nutrition, educational attainment, economic productivity, and earnings during adulthood [4].

School feeding programmes (SFPs) (also referred to as school meal programmes) are interventions that regularly provide nutritious foods (breakfast, lunch, snacks, or beverages) to children and adolescents attending school. These programmes have been well-recognised as one of the most cost-effective interventions and remain the most widespread social safety nets for children and adolescents globally, especially for those who live in areas of fragility, conflict, and violence [5]. A recent systematic review and meta-analysis examining the effects of SFPs on health and nutrition outcomes in low- and middle-income countries (LMICs) underscored their association with significant improvements in anthropometric outcomes and school attendance, but also revealed important limitations, including high heterogeneity and bias in programme design [6]. Similarly, in 2018, the Global Child Nutrition Foundation undertook a systematic landscape review to collect information on the current state of SFPs in each country, inclusive of their level of coverage (number of beneficiaries), food basket contents, and complementary programmes and services (e.g. immunisations, eye care, or water, sanitation, and hygiene (WASH)). Their findings suggest that the quantity and quality of information available on these interventions is extremely inconsistent across the 85 countries with large-scale SFPs and even across different programmes within the same country. Furthermore, information is not collected and published regularly, making it impossible to refer to the currently available information to compare school feeding operations across different settings or to discern trends over time [7]. During the COVID-19 pandemic, the World Food Programme and other relief organisations pivoted their programming by providing school meals as take-home rations and or cash transfers [8,9].

Although providing a school meal is a popular approach to improving child and adolescent diets, it does not always lead to behavioural changes [10]. As such, SFPs are often provided in conjunction with complementary health and nutrition interventions, both environmental and behavioural in nature. This approach, named ‘Health Promoting School,’ is in line with the World Health Organization’s (WHO) Global Action Plan 2013–2030 for preventing and controlling non-communicable diseases (NCDs) among school-aged children and adolescents [11]. It focusses on a broad spectrum of policies, activities, and services that contribute to the health, safety, and well-being of students, staff, and families, while assuring a supportive and healthy environment that nurtures academic growth and development [11]. This includes, but is not limited to, health services; health school environment; health and physical education; food and nutrition services, counselling; psychological and social services; health promotion for staff; and family and community involvement [11].

In LMICs, pivoting and expanding school health and nutrition programmes during crises is vital to reach at-risk children. However, there is limited synthesised literature on if and how schools adapted their policies and programming during COVID-19. In this scoping review, we aimed to synthesise available evidence on adaptation strategies, including their coverage, impact on health and nutrition outcomes in children and adolescents, and barriers and facilitators during the COVID-19 pandemic. We further provide summative guidance to stakeholders in the strategic immediate and long-term response efforts to offset the negative impacts on children and adolescents due to reduced access to vital school-based resources.

## METHODS

### Search strategy

We searched MEDLINE, Embase, and PsycINFO using a specified search strategy (Table S1 in the **Online Supplementary Document**). We included observational, intervention, programme evaluation, review, best practice, and recommendation study designs conducted in LMICs, without language restrictions. The original search was run on 15 January 2022 and updated on 16 June 2023. We also conducted pre-print and grey literature searches in several sources (Table S2 in the **Online Supplementary Document**). Ethical approval for this type of study was not required by our institute.

### Screening process and selection criteria

Two reviewers screened and selected full-text articles for review (BC, GD, and EK) and one author screened grey literature articles (KJ). Using the Joanna Briggs Institute (JBI) scoping review methodology guidance [12], we selected studies based on population, concept, context, and sources of evidence. Studies were eligible if they were published after December 2019, where the primary exposure was severe acute respiratory syndrome coronavirus 2 (SARS-CoV-2). Classification of LMICs was conducted according to the World Bank’s 2022 fiscal year country income classification. Studies that were multi-country and included both a high-income and a LMIC were required to report disaggregated data by country for inclusion (**Table 1**).

**Table 1:** Inclusion criteria

	Inclusion criteria
**Population**	School-aged children and adolescents (5–19.9 years)
**Concept**	Studies that investigated the adaptation of school health and nutrition policies and programs due to the COVID-19 pandemic. This includes adaptation strategies and components, coverage and utilisation, impact on diets, food security, anthropometry, diet-related health outcomes, and barriers and facilitators to adaptation.
**Context**	Low- and middle-income countries as defined by World Bank 2022
	Conducted in schools, virtually, in community settings, or in homes
**Sources of evidence**	Observational (i.e. cross-sectional)
	Intervention (i.e. randomised controlled trials, quasi-experimental studies)
	Programme evaluations
	Reviews
	Best practices and recommendations
**Other**	No language restrictions
	Published between January 2020 and June 2023

Studies conducted in schools, with children and adolescents (5–19.9 years) were eligible for inclusion. We used our primary outcomes of interest as eligibility criteria for including studies (i.e. we excluded a study if it did not report any of the primary outcomes). However, we did not exclude studies based on how that outcome was reported (i.e. that an effect size is not estimatable, although the outcome was clearly measured). We excluded animal studies, conference abstracts, and letters/comments, as well as those that included participants outside of the age range and did not report disaggregated data.

Studies that examined how existing school health and nutrition policies, guidelines, and programme characteristics pivoted during COVID-19 were eligible for inclusion. This included the provision of meals (breakfast, lunch, or dinner) or snacks consumed at school (in-school feeding), as well as foods distributed to the family and consumed outside of the school setting (take-home rations). We also included studies that examined food stamps or food vouchers distributed at school for the participants to access foods (in the market or food banks). Furthermore, we included both preventive and management-based health and nutrition interventions targeted at school-aged children and adolescents in LMICs which were implemented within schools before COVID-19 and adapted due to the pandemic. We considered any policy, regulation, or guideline that affected the school health and nutrition environment, including interventions that attempt to influence food availability, accessibility, policy, pricing, and promotion as an intervention.

### Data synthesis

GD, EK, and KJ extracted data independently using a standardised form to characterise and analyse the outcome data. The charted data were analysed descriptively, using tabulations or graphs where appropriate, to present a synthesis of key findings according to the scoping review objectives. The narrative synthesis of the extracted data was based on several characteristics, including geographic location of primary studies, schooling level, key components that were pivoted, and the duration of interventions. We followed the PRISMA-ScR guidelines in reporting our findings (Table S3 in the **Online Supplementary Document**).

## RESULTS

**Figure 1:**
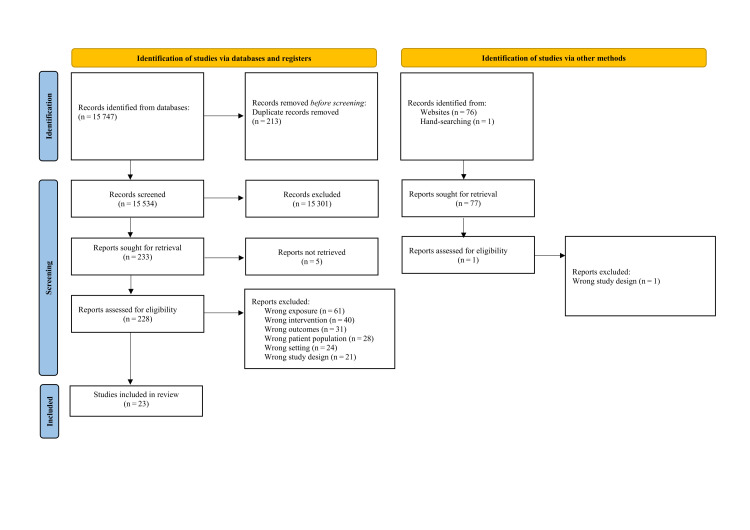
PRISMA diagram.

We identified 15 824 records in our search, of which we removed 213 through deduplication, leaving 15 611 for screening. Only 228 studies were eligible for the full-text screening stage, during which we excluded 205 studies, leaving 23 for inclusion in this review (**Figure 1** and **Table 2**).

**Table 2:** Characteristics of included studies

Author (year)	LMIC setting	Date	Study design	Population affected	Type of programme	Impact due to COVID-19
Abay et al. (2021) [13]	Nigeria	January 2019 to May 2020	Cross-sectional	Parents and children	Nutrition	Suspended indefinitely – The National Home-Grown School Feeding Program provided school meals once per day was suspended indefinitely. Found that suspension of SFP increased household food insecurity.
Chimbindi et al. (2022) [14]	South Africa	July to November 2020 and August to November 2021	Cross-sectional	Students, peer navigators, non-governmental representatives	Multi-component – nutrition and health services	Suspended indefinitely and continued in school – sexual and reproductive health education and services were suspended indefinitely while the school feeding scheme was continued in school after pandemic precautions and measures were implemented.
Colon-Ramos et al. (2021) [15]	Brazil, Chile, Colombia, Costa Rica, Ecuador, Guatemala, Mexico, Peru, Puerto Rico, Uruguay, and one subnational territory (Buenos Aires, Argentina)	April 2020 to April 2021	Mixed-methods case study (rapid assessment)	SFP managers and key informants who possessed deep knowledge about the SFP implementation	Nutrition	Administration outside schools and development of new programme – SFPs were modified to distribute food kits and/or grab-and-go meals. Rural areas of Uruguay provided food vouchers to families upon request.
Delbiso et al. (2021) [16]	Ethiopia	May 2020	Cross-sectional	Students, parents, school staff, officials from school feeding agency	Nutrition	Administration outside schools and development of new programme – the SFP providing students two meals per day was suspended indefinitely. Limited donations to the school were distributed to disadvantaged students.
Egbon et al. (2022) [17]	Nigeria	February to July 2020	Cross-sectional	Junior and senior secondary girls (nine to 19 y old)	Health services	Delayed administration – HPV vaccination programme was suspended due to COVID-19 pandemic so study team retroactively corrected disruption by returning to provide girls with the second dose of their HPV vaccines three months later than initially intended.
Francis et al. (2020) [18]	Nigeria	Not reported	Narrative review	Not reported	Multi-component – nutrition and health services	Suspended indefinitely and continued in school – SFP continued to operate in-person within schools with physical distancing while health programme (consisting of sickbays, mass deworming treatments, and annual immunizations for measles, polio, tetanus, typhoid, vitamin A, and yellow fever) was suspended indefinitely.
Hadi et al. (2021) [19]	Indonesia	January to May 2020	Mixed-methods cross-sectional	Elementary school staff representatives	Nutrition	Suspended indefinitely – access to school canteens was suspended indefinitely.
Hamoda et al. (2021) [20]	Pakistan	Not reported	Case study	Schoolchildren and teachers	Mental health	Virtual adaptation – the delivery of and training for the school mental health programme was shifted to online platforms.
Jaber et al. (2022) [21]	Lebanon	Not reported	Cross-sectional	Female school counsellors	Mental health	Virtual adaptation – school counselling for students was shifted to online platforms.
Lemes et al. (2022) [22]	Brazil	May to December 2020	Quasi-experimental study	Students (6–13 y old)	Physical activity	Virtual Adaptation – physical education classes were shifted to online platforms using Google Classroom.
Lourenco et al. (2021) [23]	Brazil	July 2020	Case study	A nutritionist and additional professional with knowledge of SFP in their municipality	Nutrition	Administration outside schools and development of New Program – SFPs in Campos dos Goytacazes were modified to distribute food kits while SFPs in Macaé were modified to provide emergency cash transfers to families.
Magis-Weinberg et al. (2021) [24]	Peru	April 2020	Case study	Students (4th and 11th grade)	Mental health	Virtual adaptation – Digital Citizenship curriculum was redesigned to a Well-Being During Lockdown curriculum that was shifted to online platforms.
Mayurasako et al. (2020) [25]	Thailand	Not reported	Viewpoint	Pre-primary and primary school students	Nutrition	Administration outside schools – SFPs were modified to distribute meals (breakfast and lunch) and shelf-stable milk.
Ntshingila et al. (2023) [26]	South Africa	September to November 2020	Cross-sectional	Social workers and nurses	Multi-component – health services and mental health	Administration outside schools – psychosocial and health screenings were conducted at home by social workers and nurses, respectively.
Prakash et al. (2021) [27]	India	Not reported	Recommendation	Students	Health services	Suspended indefinitely – School Eye Health Program was suspended indefinitely due to school closures.
Rao et al. (2022) [28]	Malaysia	2020-2022	Mini review	Female students (<15 y old)	Health services	Suspended indefinitely – HPV vaccination of female students was stopped indefinitely due to school closures.
Rawal et al. (2023) [29]	India	2019–2021	Cluster-randomised controlled trial	Elementary school students	Multi-component – nutrition and physical activity	Virtual adaptation – lifestyle curriculum, including nutrition education about healthy eating and lessons about physical activity exercises were shifted to online platforms.
Rodrigues et al. (2022) [30].	Brazil	June to July 2020	Quantitative cross-sectional	Children and their parents or guardians responsible for purchasing food	Nutrition	Administration outside schools – SFPs in Brazil were modified to distribute food baskets to local households.
Sanni et al. (2021) [31]	Nigeria	April to October 2017	Mixed-methods cross-sectional	Students from private and government-owned schools	Health Services	Stopped indefinitely – health talks and annual immunisations were stopped indefinitely due to school closures.
Sharma et al. (2021) [32]	India	June and August 2020	Research letter and government recommendation	Parents of children attending schools with the Midday Meal Scheme	Nutrition	Continued in school – although Midday Meal Scheme in India remained closed during lockdown, physical distancing measures were planned and implemented in order to continue the SFP after school re-openings.
Wang et al. (2021) [33]	China	May to October 2020	Feasibility cluster quasi-experimental study	Parent-child dyads	Physical Activity	Virtual adaptation – physical activity programme completed prior to school closure but anthropometric measurements/evaluations were retrieved virtually with the assistance of parents.
Zhang et al. (2021) [34]	China	May to July 2020	Quasi-experimental study	Students (12–18 y old)	Mixed – physical activity and mental health	Continued in-school – group psychological counselling and outdoor exercise interventions were conducted at school during the pandemic.
Zheng et al. (2021) [35]	China	March 2020	Cluster-randomised controlled trial	Secondary school students (grade 7)	Physical activity	Virtual adaptation – Chinese government-mandated recess and physical activity breaks into online homeschooling curriculum.

SFP – school food programme, HPV – human papilloma virus, y – years

Most studies were observational by design (n = 13), such as cross-sectional, cohort, and case studies [13-16,19-21,23,24,26,30,31]. Five were experimental studies, which included cluster-randomised controlled trials (n = 2) [29,35], quasi-experimental studies (QES) (n = 2) [22,34], and a feasibility cluster quasi-experimental study (n = 1) [33]. We also included a narrative review (n = 1) [18] and a mini-review (n = 1) [28]. The remaining studies (n = 3) were comprised of recommendations related to adapting school health programmes during the COVID-19 pandemic [25,27,32]. The following regions were represented across included studies: African Region (n = 7) [13,14,16-18,26,31], Region of the Americas (n = 5) [15,22–24,30], Southeast Asian Region (n = 5) [19,25,27,29,32], Western Pacific Region (n = 4) [28,33–35], and the Eastern Mediterranean Region (n = 2) [20,21] (**Table 2**).

While school-aged children remained the focus of all included studies, some investigated and allowed other populations to participate in order to provide a holistic understanding of children’s experiences both in school and at home during the COVID-19 pandemic, as well as school-level discussions that informed programmatic changes. This included primary accounts from children (n = 11) [17,18,22,24,25,27-29,31,34,35], parents (n = 1) [32], school staff such as teachers and principals (n = 1) [19], health professionals interacting with schoolchildren (n = 3) [21,23,26], or a combination of at least two of these groups (n = 7) [13-16,20,30,33].

The included studies also investigated various school programmes impacted by the COVID-19 pandemic, such as those focussed on nutrition by providing access to meals (n = 8) [13,15,16,19,23,25,30]; health services such as immunisations, eye health, and WASH (n = 4) [17,27,28,31]; physical activity curriculum and exercise training (n = 3) [22,33,35]; mental health counselling and curriculum (n = 3) [20,21,24]; or those that were multi-component (n = 5) [14,18,26,29,34]. Among the multi-component programmes, two studies looked at nutrition and health services [14,18], while one study respectively investigated health services and mental health [26], nutrition and physical activity [29], and physical activity and mental health [34].

We identified six distinct impacts due to COVID-19 across all programmes (Table S4 in the **Online Supplementary Document**). This included indefinite suspension without evidence that programmes resumed after school re-opened (n = 8) [13,14,16,18,19,27,28,31]; delayed administration that occurred after schools re-opened (n = 1) [17]; continuation of programmes in-school after the implementation of pandemic precautions and measures (n = 4) [14,18,32,34]; virtual adaptations of study components, which included the programme itself or evaluation (n = 7) [20–22,24,29,33,35]; administration of the programme outside of schools, often directly inside students’ homes (n = 5) [23,25,26,30]; or the development of a new programme in replacement (n = 3) [15,16,23].

### Multi-component programmes

The responses from multi-component programmes were found to be entirely dependent on programme type. In two studies which looked at providing nutrition and health services in South Africa and Ethiopia, respective in-school nutrition programmes continued to provide meals and supplementation during lockdowns, while their health services which comprised sexual and reproductive health education, deworming treatments, and immunisation services were suspended indefinitely [14,18]. A programme in the Ogoni area of Rivers State, Nigeria continued to operate in six schools with physical distancing by serving food to children spread across multiple classrooms, rather than a single one as was done previously. The feasibility of this was 2-fold: additional classrooms were available due to the suspension of in-person classes, while the programme only provided food to the poorest students from each school [18]. Similar to these studies, another multi-component programme for psychological counselling and outdoor exercise in China opted to continue these programmes in school to continue supporting children during the pandemic. In turn, the study found that the combination of these components led to significantly improved scores in psychological resilience among the experimental group (*P* < 0.05), although no significant differences were identified for changes in anxiety, depression, and sleep quality scores [33].

In contrast, other multi-component programmes were found to shift entirely to virtual adaptations (n = 1) or administration outside of schools (n = 1). The ‘Promoting Health Literacy in Schools Plus Study’ nutrition and physical activity intervention implemented in eight private schools across New Delhi was switched entirely to a virtual platform to promote healthy lifestyles among students, which included components such as the importance of a healthy diet; physical activity; prevention and management of NCDs, especially diabetes and obesity; and the interlinkages between NCDs and COVID-19 [29]. In comparison, psychosocial and health services screenings for students were conducted at home by social workers and nurses in South Africa as a novel government-led health promotion strategy in response to school closures [26].

### School food and nutrition programmes

Of all programme types, SFPs were found to have the most varied responses to the COVID-19 pandemic. Three studies noted programmes were entirely suspended following school closures in Nigeria, Ethiopia, and Indonesia [13,16,19,32]. The Nigerian National Home-Grown School Feeding Program, which provided one meal per day to over nine million children enrolled in government-owned primary schools (grades 1–3, ages 6–9 years), was suspended following school closures in March 2020 [13]. Similarly, the Addis Ababa city SFP, which previously provided two meals per day to approximately 360 000 students attending government primary schools, was halted indefinitely on 16 March 2020 following the closure of all schools in Ethiopia, as reported by a paper published in July 2021. It is worth noting that the decision to suspend school feeding was not due to financial constraints, as the programme already had an allocated budget per child per day; rather, the funds were to remain unused until a proven intervention mechanism was established as per the current finance regulation [16].

Moreover, school closures in Indonesia resulted in the closure of 144 school canteens among 147 surveyed public and private schools, which were still shut as of February 2021. Of these schools, 85% felt that they would be ready to re-open their canteens once provided with information regarding government regulations or guidelines for reopening, despite no such documents having been prepared at the time of this study. Readiness to re-open was 4.5 times higher among schools that owned their canteen compared to schools that did not have ownership (adjusted odds ratio (aOR) = 4.5; 95% confidence interval (CI) = 1.1, 17.9), which may be explained by greater managerial authority and control by schools with ownership [19]. The Midday Meal Scheme in India was the only programme identified that explicitly noted physical distancing measures were planned and implemented in order to continue the SFP after school re-openings [32].

To compensate for programme suspensions, four studies began distributing meals and/or food kits to the homes of students and their families [15,23,25,30]. Thirteen countries implemented this model, most of which were located in Latin America (n = 11). Food kits, which contained foods to be prepared/cooked at home, were distributed in Argentina (specifically Buenos Aires), Brazil, Chile, Columbia, Costa Rica, Ecuador, Guatemala, Mexico, and Peru [15,23,30]. Prepared meals were provided to students in Argentina (specifically Buenos Aires), Brazil, Colombia, Puerto Rico, Thailand, and Uruguay, either instead of or in addition to food kits [15,25]. Rations of specific food products were distributed in two countries. Shelf-stable milk was distributed in Thailand and grains in Uttar Pradesh State, India [25].

The frequency of distribution of food kits and meals varied by country, as well as by district and/or state within countries. In countries that specified frequency, meals were distributed more frequently than food kits, with Uruguay and Puerto Rico both providing daily meals. Food kits were distributed as frequently as every 15 days up to every month [15]. Challenges regarding the delivery of food kits and meals to students were identified as a barrier by several countries. These problems impacted food content due to refrigeration requirements and delayed distribution frequencies in some cases [15,23]. For example, the first monthly deliveries of food kits in the municipality of Campos dos Goytacazes, Brazil took approximately two to three weeks to complete [23]. In most cases, food kits and meals were expected to be picked up by students or their family members from schools or community centres. Exceptions to this included food kits that were delivered directly to students’ homes, such as in Thailand, while meals were served within schools in Uruguay despite school closures [15,25]. Moreover, seven countries reported reduced nutritional quality of food kits compared to pre-pandemic SFPs, which was attributed to insufficient financial resources and quality assurance and safety constraints of perishable foods [15,23]. Fresh products were limited and were often replaced by processed foods. Conversely, food kits in Peru contained greater diversity in the types of foods compared to those provided pre-pandemic. This was accomplished by distributing kits with foods to be prepared at home, rather than supplying processed meals as was done previously [15].

Alternatively, three papers identified several South American countries that provided financial compensation to students and their families to alleviate the burden of missed school meals [15,16,23]. Rural areas of Uruguay provided food vouchers to families upon request, which were redeemable at local supermarkets and held a value equivalent to the daily cost of a school lunch. Some areas of Colombia provided monthly food vouchers, which were valued at approximately USD 14 and could only be used to purchase certain foods [15]. While most areas of Brazil supplied meals and/or food kits, the municipality of Macaé implemented cash transfers, as this method circumvented many logistical issues associated with the distribution of food. While the Brazilian National School Feeding Program’s pre-pandemic guidelines would have prevented this, Macaé passed a municipal law in late March 2020 that authorized the payment of 200 BRL per month ( ~ 20% of the national minimum monthly wage, USD 35.03) to all students enrolled in public municipal schools. Implementation of cash transfers began in April 2020 and by August 2020; 99% of beneficiaries had received financial aid via one of three delivery models. These delivery models included: transfer of funds directly into the chequing accounts of students’ guardians; preloaded cards available for pickup at schools; and withdrawal of funds from a local bank. Despite high coverage, the authors noted that cash transfers may not be cost-effective long-term due to higher costs spent per food item compared to buying food in bulk when purchased by the municipality. Furthermore, similar to food kits, cash transfers do not guarantee that foods purchased are consumed by students, nor do they guarantee food and/or diet quality [23].

### Health services

The majority of the studies (n = 3) which investigated the impact on in-school health services found that programmes were stopped indefinitely. In Nigeria, this disruption impacted over 1450 students residing in five rural villages across the Rivers State and left them vulnerable to infectious diseases, such as measles, polio, tetanus, typhoid, and yellow fever after annual immunisations were halted [31]. Only one programme conducted in Nigeria looked to retroactively correct disruptions in human papilloma virus (HPV) vaccination among girls between nine to 14 years old. Although these girls were intended to receive their second dose in August 2020 – six months after their initial dose – the study team was unable to return to the community until November 2020 due to school closures, which extended their vaccination schedules and left five girls lost to follow-up presumably vulnerable to HPV [17].

### Physical activity

Included programmes exclusive to physical activity (n = 3) were all found to shift towards virtual adaptations. Policy-level changes were specifically implemented by the Chinese government to include virtual recess and physical activity in online schooling and education curricula. For instance, a randomised controlled trial conducted across 12 secondary schools from the Duanzhou District in Zhaoqing City aimed to investigate whether online adaptations to physical activity initiatives were able to reduce children’s anxiety, eye strain, and sleep disturbances. Students were provided with a 10-minute virtual physical activity and eye relaxation breaks in addition to 15-minute recesses four times a day. Reminders were sent through short message service prompts and exercise activities were conducted through a live-streaming application for students to remain connected with their peers. Results from this trial indicated a significant reduction in children’s anxiety from baseline between intervention and control groups (x̄ = −0.36; 95% CI = −0.63, −0.08, *P* = 0.02) and eye strain (x̄ = −0.15; 95% CI = −0.26, −0.03, *P* = 0.02) scores post-intervention [35]. Likewise, in Brazil, physical education was provided via Google Classroom to promote physical exercises, body movement practices, sports practice, dance, games, and physical activity at home, which were found to have improved students’ Self-Perceived Physical Fitness scores regarding self-perceptions of physical fitness in strength, flexibility, body weight, general fitness, and cardiorespiratory fitness [22].

### Mental health

All mental health programmes exclusively (n = 3) were found to adapt virtually. The School Mental Health Program in rural Pakistan used task-shifting to aid mental illness prevention and mental health promotion by establishing teacher-led assessment and support systems, as well as creating access to broader referral networks if needed. However, the training of teachers and student support activities were conducted through virtual chats and calls to meet the critical mental health needs of students during the pandemic. To overcome technological and financial barriers to accessing these online resources, the school curriculum was also broadcasted through televisions [20]. Likewise, five private schools in Beirut, Lebanon also adopted virtual counselling to replace traditional in-school counselling available to students. This programme had several challenges in supporting students through a virtual interface as opposed to in-school discussions that allowed for both formal and informal interactions. Counsellors mentioned virtual modalities having a steep learning curve which prompted more demanding work schedules given they were expected to be accessible for longer work hours. However, students were found to be less comfortable seeking guidance while sharing personal stories within their households, which made it difficult for counsellors to provide adequate and timely support [21].

## DISCUSSION

In this scoping review, we examined the available evidence on the adaptations of school health and nutrition programmes, including school meal programmes, health services, and physical activity education in LMICs. We found limited evidence of successful retooling of programme implementation during the pandemic-related mobility restrictions, especially from Asia and Africa. Our findings highlight the barriers school health and nutrition programmes experienced, not only in continued service provision, but also in ensuring the quality of those services once the adaptations were in place.

The most varied adaptations were in the delivery of SFPs, including their indefinite suspension or provision of alternative forms of aid, such as cash transfers. However, based on the included studies, there was an overwhelming lack of quantitative data regarding modified programme coverage, use and the impact on children, and adolescent health and nutrition. This may be attributed to limited publicly available data on national or subnational emergency responses, including by the World Food Programme and other non-governmental organisations, resources largely directed to health systems during the pandemic, and a lack of an internationally recognised framework of indicators to monitor and evaluate school health and nutrition programmes [36]. Three studies explicitly noted the impact of disruptions to SFPs on food security in Ethiopia, Nigeria, and Brazil [13,16,30]. These studies found that the suspension of the programme decreased food security and negatively impacted students’ health, whereby some students reported having skipped meals, receiving reduced portion sizes, and consuming poor-quality foods. It was also noted that the suspension of school-provided meals, in combination with parental job loss, may have reduced protections against child labour, as the need to generate income for the family becomes greater [15]. Our results are corroborated by a recent study by Ferrero et al., which gathered data from 183 programmes in 139 countries on large-scale school meal programme operations managed by countries or non-governmental organisations during COVID-19. Their results suggest that, in addition to food provisions, complementary health services significantly decreased in response to the pandemic. This not only heightens food insecurity already pervasive in low-resource settings, but also disrupts their access to health care [37].

Comprehensive school health and nutrition programmes that are tailored to country priorities and needs are an equitable and cost-effective way to improve access to health and nutrition services and play a critical role in addressing the global learning crisis [38]. Globally, 90% of countries have implemented some form of school health and nutrition programmes at scale. For example, more than 100 countries have school-based vaccination programmes, more than 450 million school-age children are dewormed every year in schools in LMICs, and almost every country includes education for health and well-being in its curriculum [38]. School feeding programmes offer healthy meals, which are critical for nutritionally vulnerable school-aged children and adolescents. These programmes are particularly cost-effective because they deliver returns across multiple sectors, including education, health, agriculture, and social protection, with US 9 in returns for every US 1 invested [39]. Psychosocial programmes that address anxiety, depression, and suicide can provide an average return on investment across all countries of US 21.5 for every US 1 invested over 80 years [40].

Importantly, the COVID-19 pandemic has provided a unique opportunity to evaluate current gaps in programming and build back better [41]. In 2021, governments united to acknowledge the necessity for an innovative approach to supporting school-aged children and adolescents while simultaneously promoting sustainable dietary habits and food systems. This culmination led to the introduction of a global School Meals Coalition at the United Nations Food System Summit in October 2021 [9]. Currently, this coalition consists of 76 member states, collectively responsible for 58% of the world's student population, representing diverse geographical regions and encompassing high, middle, and low-income nations [9]. The creation of financially independent, intersectoral working groups may be useful to provide guidelines on best practices for service delivery during school closures to ensure effective usage of finite resources [15].

While globally, most countries have policies related to school health and nutrition [9], in order to be resilient to emergencies and crises, health and nutrition adaptation policies must be institutionalised in education systems by establishing dedicated committees tasked with developing and implementing such policies at national, subnational, and local levels. According to joint guidance provided by the Food and Agriculture Organization, World Food Programme, and UNICEF, countries should establish a multisectoral emergency task force that is responsible for school feeding, either as part of a broader response to the food and nutrition situation or independently, to rapidly assess the situation and provide options of feasible responses is recommended [42]. Of the 11 identified Latin American countries in this review, there was no mention of SFPs in the emergency declarations of any country, except for a general reference to feeding made by Peru. Due to lack of established guidelines, many countries struggled early on to determine the most effective strategies to continue providing meals to students. In Chile, Colombia, Ecuador, Guatemala, and Brazil, SFPs are protected by national law, which enabled mobilisation of resources, helped to maintain nutritional standards, and provided political support for the continuation of modified SFPs during the pandemic [15]. For example, Brazilian National School Nourishment Program guidelines require that municipalities continue to ensure students’ right to adequate food even in times of crisis [23,30]. However, legislation and guidelines in several countries required modification to make them more suited for the changing needs of programmes during the pandemic [15]. Before the pandemic, the guidelines stipulated that meals could only be offered to students at schools. This changed on 7 April 2020, when the Ministry of Education passed a federal law (Federal Law No.13 987) that allowed municipalities to distribute food directly to students or their parents/guardians during an emergency [23]. Changes to SFP policies were not as flexible in other countries, which was also identified as a barrier. Notably, legislation in Peru (D.Leg. No. 1472) restricted schools’ financial autonomy needed to modify SFPs [15].

Besides policies, school infrastructure and resources should align with international standards and guidelines by ensuring proper ventilation, WASH facilities, barrier fencing for traffic control, and social distancing [31]. The pandemic has already prompted the use of virtual platforms, which has been shown to provide some sustainability to school physical and mental health programmes [35]. Investing in similar technological resources to maintain access to health services in other contexts may be valuable to ensuring children’s health and well-being. Alternatively, school health programmes that may require in-person consultations, such as food provisions, immunisations, and treatments may benefit from collaborating with existing community facilities. For instance, providing school food provisions through food banks and other community safety net programmes has been encouraged to curb the massive impact of lost meals amongst children in Ethiopia [16]. Conducting immunisations and treatments within local clinics and primary care facilities may be a similar adaptation worth exploring to ensure children and their families are still able to access vital health services during emergency crises that mandate school closures.

Although this was an exploratory review, it highlights the lack of evidence on school health and nutrition programming during a global pandemic. School health and nutrition programmes should be monitored under normal circumstances and during times of crisis in order to accurately measure the success of modified programmes and to support evidence-based policy and programme development. While the adoption of the WHO global standards and indicators is necessary at the national and school level, future research must prioritise the development of comprehensive school monitoring and evaluation framework to track key indicators related to both the health and nutrition of school-aged children and adolescents. Programme monitoring should be integrated into existing school-based data sources and should promote the flow of information from the local to national levels [32]. The collection of and access to sex- and gender-disaggregated data has also been limited, which has made it difficult to determine the gendered impacts of COVID-19 and, in turn, the disruptions in school health and nutrition programmes. Therefore, ensuring the inclusion of gender-specific data into existing monitoring and evaluation systems is crucial to informing the development of future policies and programmes [43].

## CONCLUSIONS

Schools across the globe experienced prolonged and intermittent closures during the COVID-19 pandemic, with the most recent of these occurring as recently as March 2022. The adverse effects of these school closures range from loss in learning, premature school dropouts (especially for girls), as well as poorer mental health and social development of school-aged children, potentially translating into long-term adverse outcomes into adulthood. The recommendations highlighted in our review apply to not only pre-existing programmes aiming to build back better, and gain the ground lost over the last three years, but also provide a framework to be used in times of future crises, whether it be an infectious diseases epidemic or pandemic, conflict or natural disaster. If we are to learn one thing from the COVID-19 pandemic and its impact on the health and well-being of children and adolescents, it is that continued provision of educational, health and nutrition services should be of paramount concern for stakeholders and policymakers across all sectors and levels of governments.

## Additional material


Online Supplementary Document
