# Supplementary material for: Pivoting school health and nutrition programmes during COVID-19 in low- and middle-income countries: A scoping review
Source: J Glob Health. 2024 Jan 19;14:05006. doi: 10.7189/jogh.14.05006 (PMC10795858; doi:10.7189/jogh.14.05006)
Supplement: Online Supplementary Document [file jogh-14-05006-s001.pdf]

Supplementary File

Table S1. Search Strategy for MEDLINE

MEDLINE (Ovid)		
Search Number	Search Terms	Results
1	Exp Child/	2041257
2	Exp Adolescent/	2151191
3	Exp Students/	149310
4	Exp Schools/	133871
5	(child* or minor* or adolescen* or teen* or youth* or young* or kid* or schoolgirl* or school-girl* or schoolboy* or school-boy* or schoolchild* or school-child* or schoolage* or school-age* or student* or pupil*).ti,ab,sh,kf.	5826215
6	(school* or primary school* or elementary school* or middle school* or secondary school* or high school* or kindergarten*).ti,ab,sh,kf.	378729
7	Exp Child Health/	4136
8	Exp Adolescent Health/	1656
9	Exp Child Health Services/	25320
10	Exp Adolescent Health Services/	5812
11	Exp Preventive Health Services/	640016
12	Exp Health Education/	256085
13	Exp Health Policy/	112211
14	Exp Food Assistance/	1518
15	(school meal program* or school breakfast program* or school lunch program* or school snack program* or school feed* program* or school food program* or school nutrition program* or student meal program* or student breakfast program* or student lunch program* or student snack program* or student feed* program* or student food program* or student nutrition program* or school health program* or student health program* or food aid* or food assistance).ti,ab,sh,kf.	3763
16	(school* adj4 program*).ti,ab,sh,kf.	20698
17	(school* adj4 promotion).ti,ab,sh,kf.	1379
18	(school* adj4 polic*).ti,ab,sh,kf.	3724
19	(school* adj4 guideline*).ti,ab,sh,kf.	1459
20	(school* adj4 service*).ti,ab,sh,kf.	5039
21	(school* adj4 intervention*).ti,ab,sh,kf.	8267
22	(school* adj4 regulat*).ti,ab,sh,kf.	441

23	(afghanistan OR albania OR algeria OR american samoa OR angola OR "antigua and barbuda" OR antigua OR barbuda OR argentina OR armenia OR armenian OR aruba OR azerbaijan OR bahrain OR bangladesh OR barbados OR republic of belarus OR belarus OR byelarus OR belorussia OR byelorussian OR belize OR british honduras OR benin OR dahomey OR bhutan OR bolivia OR "bosnia and herzegovina" OR bosnia OR herzegovina OR botswana OR bechuanaland OR brazil OR brasil OR bulgaria OR burkina faso OR burkina fasso OR upper volta OR burundi OR urundi OR cabo verde OR cape verde OR cambodia OR kampuchea OR khmer republic OR cameroon OR cameron OR cameroun OR central african republic OR ubangi shari OR chad OR chile OR china OR colombia OR comoros OR comoro islands OR iles comores OR mayotte OR democratic republic of the congo OR democratic republic congo OR congo OR zaire OR costa rica OR "cote d'ivoire" OR "cote d' ivoire" OR cote divoire OR cote d ivoire OR ivory coast OR croatia OR cuba OR cyprus OR czech republic OR czechoslovakia OR djibouti OR french somaliland OR dominica OR dominican republic OR ecuador OR egypt OR united arab republic OR el salvador OR equatorial guinea OR spanish guinea OR eritrea OR estonia OR eswatini OR swaziland OR ethiopia OR fiji OR gabon OR gabonese republic OR gambia OR "georgia (republic)" OR georgian OR ghana OR gold coast OR gibraltar OR greece OR grenada OR guam OR guatemala OR guinea OR guinea bissau OR guyana OR british guiana OR haiti OR hispaniola OR honduras OR hungary OR india OR indonesia OR timor OR iran OR iraq OR isle of man OR jamaica OR jordan OR kazakhstan OR kazakh OR kenya OR "democratic people's republic of korea" OR republic of korea OR north korea OR south korea OR korea OR kosovo OR kyrgyzstan OR kirghizia OR kirgizstan OR kyrgyz republic OR kirghiz OR laos OR lao pdr OR "lao people's democratic republic" OR latvia OR lebanon OR lebanese republic OR lesotho OR basutoland OR liberia OR libya OR libyan arab jamahiriya OR lithuania OR macau OR macao OR republic of north macedonia OR macedonia OR madagascar OR malagasy republic OR malawi OR nyasaland OR malaysia OR malay federation OR malaya federation OR maldives OR indian ocean islands OR indian ocean OR mali OR malta OR micronesia OR federated states of micronesia OR kiribati OR marshall islands OR nauru OR northern mariana islands OR palau OR tuvalu OR mauritania OR mauritius OR mexico OR moldova OR moldovian OR mongolia OR montenegro OR morocco OR ifni OR mozambique OR portuguese east africa OR myanmar OR burma OR namibia OR nepal OR netherlands antilles OR nicaragua OR niger OR nigeria OR oman OR muscat	2224684

	OR pakistan OR panama OR papua new guinea OR new guinea OR paraguay OR peru OR philippines OR philipines OR phillipines OR phillippines OR poland OR "polish people's republic" OR portugal OR portuguese republic OR puerto rico OR romania OR russia OR russian federation OR ussr OR soviet union OR union of soviet socialist republics OR rwanda OR ruanda OR samoa OR pacific islands OR polynesia OR samoan islands OR navigator island OR navigator islands OR "sao tome and principe" OR saudi arabia OR senegal OR serbia OR seychelles OR sierra leone OR slovakia OR slovak republic OR slovenia OR melanesia OR solomon island OR solomon islands OR norfolk island OR norfolk islands OR somalia OR south africa OR south sudan OR sri lanka OR ceylon OR "saint kitts and nevis" OR "st. kitts and nevis" OR saint lucia OR "st. lucia" OR "saint vincent and the grenadines" OR saint vincent OR "st. vincent" OR grenadines OR sudan OR suriname OR surinam OR dutch guiana OR netherlands guiana OR syria OR syrian arab republic OR tajikistan OR tadjikistan OR tadzhikistan OR tadzhik OR tanzania OR tanganyika OR thailand OR siam OR timor leste OR east timor OR togo OR togolese republic OR tonga OR "trinidad and tobago" OR trinidad OR tobago OR tunisia OR turkey OR turkmenistan OR turkmen OR uganda OR ukraine OR uruguay OR uzbekistan OR uzbek OR vanuatu OR new hebrides OR venezuela OR vietnam OR viet nam OR middle east OR west bank OR gaza OR palestine OR yemen OR yugoslavia OR zambia OR zimbabwe OR northern rhodesia OR global south OR africa south of the sahara OR sub-saharan africa OR subsaharan africa OR africa, central OR central africa OR africa, northern OR north africa OR northern africa OR magreb OR maghrib OR sahara OR africa, southern OR southern africa OR africa, eastern OR east africa OR eastern africa OR africa, western OR west africa OR western africa OR west indies OR indian ocean islands OR caribbean OR central america OR latin america OR "south and central america" OR south america OR asia, central OR central asia OR asia, northern OR north asia OR northern asia OR asia, southeastern OR southeastern asia OR south eastern asia OR southeast asia OR south east asia OR asia, western OR western asia OR europe, eastern OR east europe OR eastern europe OR developing country OR developing countries OR developing nation? OR developing population? OR developing world OR less developed countr* OR less developed nation? OR less developed population? OR less developed world OR lesser developed countr* OR lesser developed nation? OR lesser developed population? OR lesser developed world OR under developed countr* OR under developed nation? OR under developed population? OR under developed world OR	

	underdeveloped countr* OR underdeveloped nation? OR underdeveloped population? OR underdeveloped world OR middle income countr* OR middle income nation? OR middle income population? OR low income countr* OR low income nation? OR low income population? OR lower income countr* OR lower income nation? OR lower income population? OR underserved countr* OR underserved nation? OR underserved population? OR underserved world OR under served countr* OR under served nation? OR under served population? OR under served world OR deprived countr* OR deprived nation? OR deprived population? OR deprived world OR poor countr* OR poor nation? OR poor population? OR poor world OR poorer countr* OR poorer nation? OR poorer population? OR poorer world OR developing econom* OR less developed econom* OR lesser developed econom* OR under developed econom* OR underdeveloped econom* OR middle income econom* OR low income econom* OR lower income econom* OR low gdp OR low gnp OR low gross domestic OR low gross national OR lower gdp OR lower gnp OR lower gross domestic OR lower gross national OR lmic OR lmics OR third world OR lami countr* OR transitional countr* OR emerging economies OR emerging nation?).mp	
24	(afghan OR afghans OR afghani OR albanian? algerian? OR american samoan? OR angolan? OR antiguan? OR barbudan? OR argentine? OR argentinian? OR argentinean? OR armenian? OR aruban? OR azerbaijani? OR bahraini? OR bangladeshi? OR bangalees OR bajan? OR belarusian? OR byelorussian? OR belizean? OR beninese? OR bhutanese OR bolivian? OR bosnian? OR botswana OR batswana OR brazilian? OR brasilian? OR bulgarian? OR burkinabe OR burkinese OR burundian? OR cape verdean? OR cabo verdean? OR cambodian? OR khmer OR cameroonian? OR central african? OR chadian? OR chilean? OR chinese OR colombian? OR comorian? OR congolese OR costa rican? OR ivorian? OR croatian? OR cuban? OR cypriot? OR czech? OR djiboutian? OR dominican? OR ecuadorian? OR egyptian? OR salvadoran? OR equatorial guinean? OR equatoguinean? OR eritrean? OR estonian? OR swazi? OR swati? OR ethiopian? OR fijian OR gabonese OR gabonaise OR gambian? OR georgian? OR ghanaian? OR gibraltarian? OR greek? OR grenadian? OR guamanian? OR guatemalan? OR guinean? OR bissau guinean? OR guyanese OR haitian? OR honduran? OR hungarian? OR indian? OR indonesian? OR iranian? OR iraqian? OR iraqi? OR manx OR jamaican? OR jordanian? OR kazakhstani? OR kenyan? OR kirabati OR kirabatian? OR north korean? OR korean? OR kosovar? OR kosovan? OR kyrgyz* OR lao OR	950820

	laotian? OR latvian? OR lebanese OR lesothan? OR lesothonian? OR mosotho OR basotho OR liberian? OR libyan? OR lithuanian? OR macanese OR macedonian? OR malagasy OR madagascan? OR malawian? OR malaysian? OR maldivian? OR malian? OR maltese OR marshallese? OR mauritanian? OR mauritian? OR mexican? OR micronesian? OR moldovan? OR mongolian? OR mongol OR montenegrin? OR moroccan? OR mozambican? OR burmese OR myanma OR namibian? OR nauruan? OR nepali OR nepalese OR netherlands antillean? OR nicaraguan? OR nigerien? OR nigerian? OR northern mariana islander? OR mariana? OR omani? OR pakistani? OR palauan? OR panamanian? OR papua new guinean? OR paraguayan? OR peruvian? OR philippine? OR philipine? OR phillipine? OR phillippine? OR filipino? OR filipina? OR polish OR pole OR poles OR portuguese OR puerto rican? OR romanian? OR russian? OR soviet people OR soviet population OR rwandan? OR rwandese OR ruandan? OR ruandese OR samoan? OR sao tomean? OR santomean? OR saudi arabian? OR saudi? OR senegalese OR serbian? OR montenegrin? OR seychellois OR seychelloise? OR sierra leonean? OR slovak? OR slovene? OR solomon islander? OR somali? OR south african? OR south sudanese OR sri lankan? OR ceylonese OR kittitian? OR nevisian? OR saint lucian? OR vincentian? OR sudanese OR surinamese? OR syrian? OR tajik? OR tajikistani? OR tanzanian? OR tanganyikan? OR thai OR timorese? OR togolese OR tongan? OR trinidadian? OR tobagonian? OR tunisian? OR turk? OR turkish OR turkmen? OR tuvaluan? OR ugandan? OR ukrainian? OR uruguayan? OR uzbek? OR vanuatu* OR venezuelan? OR vietnamese OR yemeni? OR yemenite? OR yemenese OR yugoslav? OR yugoslavian? OR zambian? OR zimbabwean?).ti,ab,sh,kf.	
25	1 or 2 or 3 or 4 or 5 or 6	5933707
26	7 or 8 or 9 or 10 or 11 or 12 or 13 or 14 or 15 or 16 or 17 or 18 or 19 or 20 or 21 or 22	789292
27	23 or 24	2624465
28	25 and 26 and 27	65554
29	Limit 28 to 2020-Current	7056

Table S2: Grey Literature Searches

World Food Programme
Food and Agriculture Organization
UNICEF
UNICEF Innocenti
UNESCO
UNDP
UNFPA
World Bank
3ie
WHO
African Development Bank
Asian Development Bank
Global Child Nutrition Foundation
World Vision
Child-to-Child
Centre for Global Development
UN Research Institute for Social Development
UN Human Development Reports
Emergency Nutrition Network
Avenir Health
Open Grey
Children's Investment Fund Foundation
J-PAL
Data 2X
Young Lives
GAGE

Table S3. Preferred Reporting Items for Systematic reviews and Meta-Analyses extension for Scoping Reviews (PRISMA-ScR) Checklist

SECTION	ITEM	PRISMA-ScR CHECKLIST ITEM	REPORTED ON PAGE #
**TITLE**			
Title	1	Identify the report as a scoping review.	Cover Page
**ABSTRACT**			
Structured summary	2	Provide a structured summary that includes (as applicable): background, objectives, eligibility criteria, sources of evidence, charting methods, results, and conclusions that relate to the review questions and objectives.	Page 1
**INTRODUCTION**			
Rationale	3	Describe the rationale for the review in the context of what is already known. Explain why the review questions/objectives lend themselves to a scoping review approach.	Page 2
Objectives	4	Provide an explicit statement of the questions and objectives being addressed with reference to their key elements (e.g., population or participants, concepts, and context) or other relevant key elements used to conceptualize the review questions and/or objectives.	Page 3
**METHODS**			
Protocol and registration	5	Indicate whether a review protocol exists; state if and where it can be accessed (e.g., a Web address); and if available, provide registration information, including the registration number.	Not completed
Eligibility criteria	6	Specify characteristics of the sources of evidence used as eligibility criteria (e.g., years considered, language, and publication status), and provide a rationale.	Page 3-4, Table 1
Information sources*	7	Describe all information sources in the search (e.g., databases with dates of coverage and contact with authors to identify additional sources), as well as the date the most recent search was executed.	Page 3
Search	8	Present the full electronic search strategy for at least 1 database, including any limits used, such that it could be repeated.	Page 3
Selection of sources of evidence†	9	State the process for selecting sources of evidence (i.e., screening and eligibility) included in the scoping review.	Page 3-4

SECTION	ITEM	PRISMA-ScR CHECKLIST ITEM	REPORTED ON PAGE #
Data charting process‡	10	Describe the methods of charting data from the included sources of evidence (e.g., calibrated forms or forms that have been tested by the team before their use, and whether data charting was done independently or in duplicate) and any processes for obtaining and confirming data from investigators.	Page 4
Data items	11	List and define all variables for which data were sought and any assumptions and simplifications made.	Not completed
Critical appraisal of individual sources of evidence§	12	If done, provide a rationale for conducting a critical appraisal of included sources of evidence; describe the methods used and how this information was used in any data synthesis (if appropriate).	Not completed
Synthesis of results	13	Describe the methods of handling and summarizing the data that were charted.	Page 4
**RESULTS**			
Selection of sources of evidence	14	Give numbers of sources of evidence screened, assessed for eligibility, and included in the review, with reasons for exclusions at each stage, ideally using a flow diagram.	Figure 1/Page 4
Characteristics of sources of evidence	15	For each source of evidence, present characteristics for which data were charted and provide the citations.	Table 2/Page 4
Critical appraisal within sources of evidence	16	If done, present data on critical appraisal of included sources of evidence (see item 12).	Not completed
Results of individual sources of evidence	17	For each included source of evidence, present the relevant data that were charted that relate to the review questions and objectives.	Pages 4-10
Synthesis of results	18	Summarize and/or present the charting results as they relate to the review questions and objectives.	Pages 4-10
**DISCUSSION**			
Summary of evidence	19	Summarize the main results (including an overview of concepts, themes, and types of evidence available), link to the review questions and objectives, and consider the relevance to key groups.	Pages 11-12
Limitations	20	Discuss the limitations of the scoping review process.	Pages 11-12

SECTION	ITEM	PRISMA-ScR CHECKLIST ITEM	REPORTED ON PAGE #
Conclusions	21	Provide a general interpretation of the results with respect to the review questions and objectives, as well as potential implications and/or next steps.	Page 12-13
**FUNDING**			
Funding	22	Describe sources of funding for the included sources of evidence, as well as sources of funding for the scoping review. Describe the role of the funders of the scoping review.	Cover Page

JBI = Joanna Briggs Institute; PRISMA-ScR = Preferred Reporting Items for Systematic reviews and Meta-Analyses extension for Scoping Reviews.

* Where *sources of evidence* (see second footnote) are compiled from, such as bibliographic databases, social media platforms, and Web sites.

† A more inclusive/heterogeneous term used to account for the different types of evidence or data sources (e.g., quantitative and/or qualitative research, expert opinion, and policy documents) that may be eligible in a scoping review as opposed to only studies. This is not to be confused with *information sources* (see first footnote).

‡ The frameworks by Arksey and O'Malley (6) and Levac and colleagues (7) and the JBI guidance (4, 5) refer to the process of data extraction in a scoping review as data charting.

§ The process of systematically examining research evidence to assess its validity, results, and relevance before using it to inform a decision. This term is used for items 12 and 19 instead of "risk of bias" (which is more applicable to systematic reviews of interventions) to include and acknowledge the various sources of evidence that may be used in a scoping review (e.g., quantitative and/or qualitative research, expert opinion, and policy document).

Table S4. Adaptation Strategies by Program Implemented in Low- and Middle- Income Countries During COVID-19

Impact Due to COVID	Type of Program					
	Multi-Component (n=5)*	Nutrition (n=10)	Health Services (n=4)	Physical Activity (n=3)	Mental Health (n=3)	TOTAL *
Suspended Indefinitely	2	3	3			8
Delayed Administration			1			1
Continued In-School	3	1				4
Virtual Adaptation	1			3	3	7
Administration Outside Schools	1	4				5
Development of New Program		3				3

***Note: Some studies may be represented in in multiple columns for two reasons: (1) multi-component programs may have pursued different adaptation methods for their respective components (i.e., stopped health services entirely but continued food programs in-school with pandemic precautions) or (2) singular programs may have pursued multiple adaptation methods to maximize outreach during the COVID-19 pandemic (i.e., meals were administered to homes while a cash transfer program was also developed for student households).**
